# Effect of Juvenile Hormone on Worker Behavioral Transition in the Red Imported Fire Ant, *Solenopsis invicta* (Hymenoptera: Formicidae)

**DOI:** 10.3390/insects14120934

**Published:** 2023-12-08

**Authors:** Qilin Ren, Lin Ma, Xiaolong Zhang, Libiao Chen, Zhigang Mao, Dongdong Li, Lei Zhang, Xingfu Jiang

**Affiliations:** 1 State Key Laboratory for Biology of Plant Diseases and Insect Pests, Institute of Plant Protection, Chinese Academy of Agricultural Science, Beijing 100193, China; qlren94@163.com (Q.R.); linma1990@163.com (L.M.); 82101235361@caas.cn (X.Z.); 2 Guangxi Green City Pest Control Technology Co., Ltd., Nanning 530007, China; clb5125@163.com; 3 Guangxi Beitou Urban Environmental Governance Group Co., Ltd., Nanning 530000, China; jtmzg@126.com (Z.M.); ldd811217@126.com (D.L.)

**Keywords:** juvenile hormone, *Solenopsis invicta*, social insects, nurse worker, behavioral transition, brood care behavior, phototaxis

## Abstract

**Simple Summary:**

The division of labor among workers is one of the predominant characteristics of social insects, which simply means that workers perform different tasks, such as caring for the brood inside the nest and foraging outside the nest, and which is essential for colony maintenance and development. Previous studies on honeybees have demonstrated that the regulation of behavioral transitions can be influenced by juvenile hormone (JH). The red imported fire ant (RIFA), *Solenopsis invicta*, is a typical social pest that poses a significant risk to biodiversity, ecosystems, and public health in invaded areas. Understanding the effects of JH on the behavioral transitions of RIFA is necessary, as it may provide valuable experimental data for pest control. In this study, we simulated JH elevation using the juvenile hormone analogue (JHA) methoprene application and evaluated its effect on activity levels, brood care behavior, phototaxis, and threat responsiveness of nurse workers. Our study indicates that the application of JHA reduced brood care behavior and enhanced phototaxis in nurse workers, thereby revealing the role of JH in facilitating behavioral transitions in RIFA from intranidal tasks to extranidal activity. These findings may contribute to a better understanding of the underlying mechanisms behind the division of labor in social insects.

**Abstract:**

The division of labor among workers is a defining characteristic of social insects and plays a pivotal role in enhancing the competitive advantage of their colony. Juvenile hormone (JH) has long been hypothesized to be the essential driver in regulating the division of labor due to its ability to accelerate behavioral transitions in social insects, such as honeybees. The regulation of behavioral transitions by JH in the red imported fire ant (RIFA), *Solenopsis invicta*, a typical social pest, is unclear. Through video capture and analysis, we investigated the effects of the juvenile hormone analogue (JHA) methoprene on brood care, phototaxis behavior, and threat responsiveness of RIFA nurse workers. Our results showed that the JHA application significantly reduced the time and frequency of brood care behavior by nurse workers while increasing their walking distance and activity time in the light area. Additionally, the application of JHA made ants become excited, indicating a significant improvement in their activity level (movement distance, time, and speed). Furthermore, it was observed that the application of JHA did not affect the threat responsiveness of nurse workers towards stimuli (nestmates or non-nestmates). Our study demonstrates that the application of JHA reduced brood care behavior and enhanced phototaxis in nurse workers, which may reveal the role of JH in facilitating behavioral transitions in RIFA from intranidal tasks to extranidal activity. This study provides an experimental basis for further elucidating the mechanism underlying the division of labor in social insects.

## 1. Introduction

Under the influence of external environmental or intra-colony selection pressure, social insects often exhibit altruistic behavior at the group level through the division of labor among different individuals [1]. The division of labor in social insects generally involves the division of labor between reproductive and non-reproductive individuals, with the colony typically consisting of a few reproductive caste individuals and the majority being non-reproductive individuals. Parental care and reproductive division are the predominant characteristics of typical social insects, such as bees, ants (Hymenoptera), and termites (Isoptera) [2,3]. The division of labor is not only a characteristic of social insect species but is also key to achieving social dominance [4]. Individuals of different castes exhibit distinct behaviors due to the division of labor. Adult workers of most social insect species perform diverse tasks, such as brooding care inside the nest, foraging outside the nest, and building and protecting the nest. These behaviors are essential for maintaining and developing the colony [2,5]. The division of labor based on morphology and age of adult workers is defined as caste and temporal polyethism, respectively [6]. Age-based temporal polyethism is more common in social insects, with young workers nursing in nests and older workers foraging for the colony [2]. However, the division of labor among workers is to some extent flexible, rather than constant [5]. Understanding the mechanisms of behavioral differentiation and division of labor in social insects is one of the crucial issues in the field of social insect biology and behavior.

Juvenile hormone (JH) plays a crucial role in the development and reproduction of insects, and its regulatory role in determining the division of labor among social insects has also been extensively studied [7,8,9,10,11]. The changes in JH levels in honeybee larvae, resulting from being fed different nutritional foods, affect the caste differentiation of their adult bees (developing into queen bees or worker bees) [12]. JH is also involved in regulating age polyethism and the propensity for behavioral tasks in adult workers [9,13,14]. Among several social insect species, such as *Polistes canadensis*, *Polistes dominulus*, *Pogonomyrmex californicus*, *Myrmicaria eumenoides*, and *Harpegnathos saltator*, the JH level of foraging workers is higher than that of nest workers [15,16,17,18,19]. Topical application of a juvenile hormone analogue (JHA) to leaf-cutting ants resulted in an increase in JH titer, which induced a behavioral transition among workers and led them to engage in more extranidal activity instead of intranidal work [20].

The red imported fire ant (RIFA), *Solenopsis invicta*, is a globally high-risk invasive pest that poses a major threat to native biodiversity, ecosystems, and public property and security of the invaded areas [21,22]. As a typical social insect, adult RIFA workers are categorized based on their division of labor as nurse, reserve, and forager workers. Nurse workers are responsible for feeding and grooming larvae and queens, sensing changes in temperature and humidity within the nest, and transporting the larvae and queens to appropriate locations [23], while forager workers tend toward protecting the nest and extranidal activities [21,24]. The high social division of labor in the RIFA facilitates their invasion and spread, while increasing the difficulty of pest control. However, it remains to be verified whether the level of JH affects the social behavior of the RIFA.

In this study, we evaluated the effect of JHA application on the behavior of RIFA nurse workers. Video was taken to record and analyze the effects of continuous feeding of JHA methoprene on the movement level, brood care behavior, phototaxis, and threat responsiveness of nurse ants. Our results will contribute to the understanding of the division of labor mechanism of social insects and provide the experimental basis for the application of JH in the prevention and control of RIFA.

## 2. Materials and Methods

### 2.1. Ant Colonies

Colonies of the RIFA were collected from the field in Wuling Village (22.966194° N, 108.091219° E), located in Nanning, Guangxi Province, China. Eight separate colonies, collected from various locations, were transported to the laboratory. The colonies were kept in plastic containers (60 cm × 42 cm × 20 cm) with Fluon coating on the inner walls, and black shaded containers (20 cm × 12 cm × 5cm) were provided as artificial nests. The colonies were fed twice a week with mealworms, *Tenebrio molitor*, and a 10% honey–water solution (*w*/*w*), along with an adequate water supply. The colonies were maintained at 26 ± 2 °C with 12:12 h light/dark photoperiod under a relative humidity of 60%. A laboratory-adapted colony was randomly chosen for subsequent tests.

### 2.2. Selection of Experimental Fire Ant Workers

The division of labor among worker ants may be related to the spatial location inside the nest [25,26]. The RIFA workers were classified into three groups based on their division of labor: nurses, reserves, and foragers [24]. Those that remained in the nest to care for the brood were considered nurses. The method used to distinguish nurse ants from foragers followed the approach outlined by Chen et al. [27]. The worker ants that appeared in the food area were identified as foragers. Thus, the worker ants that remained in the artificial nest covered with brood were selected as nurses. The head width of worker ants serves as a crucial indicator of their body size [28], which in turn significantly influences the stability of labor division [29,30]. In this study, to control for the influence of body size on the labor division of workers, medium-sized nurses with a head width ranging from 0.6 to 1 mm were selected for follow-up experiments.

### 2.3. Applications of the Juvenile Hormone Analogue

Methoprene (provided by Cato Research Chemicals Inc., Eugene, OR, USA, with a purity of 94.7%) was chosen as the juvenile hormone analogue (JHA) for the experiment. Methoprene was added into pure water and thoroughly mixed using a vortex mixer (OSE-VX-01, Tiangen Biotech (Beijing) Co., Ltd., Beijing, China) to obtain a 4mg/mL aqueous suspension of methoprene. Previous studies have proven that feeding the approximate dose of methoprene has physiological and behavioral effects on social insects, such as *Apis mellifera* and *Pogonomyrmex rugosus* [31,32]. Medium-sized nurses fed with either the newly prepared methoprene aqueous suspension or pure water were used as the JHA treatment group and the control group, respectively. For each group, two hundred medium-sized nurse ants were collected from the same colony and individually placed in round plastic boxes (with a base diameter of 13 cm and a height of 7 cm) with Fluon coating on their inner walls. Sterile cotton balls saturated with newly prepared methoprene aqueous suspension or pure water were provided to feed the medium-sized nurse ants, with daily changes and continuous feeding for 14 days. The survival of nurse ants was observed and recorded daily, with deceased worker ants subsequently removed from the plastic boxes. After 14 days of feeding treatment, the surviving medium-sized nurses from the JHA treatment group and control group were used for subsequent behavioral experiments.

### 2.4. Assessment of Brood Care Behavior

Brood care behavior, such as cleaning, guarding, or touching the brood, is one of the important labor divisions of nurse ants. The assessment of brood care behavior was modified from the method of Pamminger et al. [33]. A circular plastic chamber (with a bottom diameter of 20 mm and a height of 2 mm) with a transparent cover plate was designed and used to observe and measure the brood care behavior of single-headed medium-sized nurses from the JHA treatment group and the control group. Single pupa from the same colony were affixed to one side of a circular plastic chamber using a non-toxic and odorless aqua-solvable environmental protective glue (deli, Ningbo, China). All pupae selected for the study were of comparable size, with a body length from 2 to 3 mm. A single nurse worker was given 10 min to acclimate to the conditions within the circular plastic chamber before recording. Subsequently, a camera (USB4KHDR01-V100, Quanrui, China) fixed directly above the circular plastic chamber was used to continuously capture video for 30 min. The video was recorded in darkness to simulate the absence of light inside the nest. Fifty nurse ants were assigned to each treatment group. We used the EthoVision XT (v17.5) software (Noldus Information Technology, Wageningen, The Netherlands) to analyze the video and obtain the total movement distance, total movement time, and average movement speed of the nurse ants [27,34]. We drew a circular area with a diameter of 3 mm centered on the pupa. The number of times nurse ants entered and exited this circular area during observation was recorded as brood care frequency, while the total time spent by nurse ants in this circular area was recorded as total brood care time.

### 2.5. Assessment of Phototactic Behavior

The assessment of phototactic behavior was modified from the method of Norman and Hughes [20]. A circular plastic chamber (with a bottom diameter of 30 mm and a height of 2 mm) with a transparent cover plate was designed and used to observe and measure the phototactic behavior of single-headed medium-sized nurses from the JHA treatment group and the control group. The cover plate of each circular plastic chamber was shaded with black tape to form a half-light and half-dark observation room. A single nurse worker was given 5 min to acclimate to the conditions within the circular plastic chamber before recording. Subsequently, a camera (USB4KHDR01-V100, Quanrui, China) fixed directly above the circular plastic chamber was used to continuously capture video for 10 min. Sixty nurse ants were assigned to each treatment group. We used the EthoVision XT (v17.5) software to analyze the video and obtain the total movement distance and total movement time of the nurse ants spent in the half-light zone.

### 2.6. Assessment of Threat Responsiveness

The method of the mandible opening response (MOR) assay described in Norman et al. [35] was used to assess the threat responsiveness of the nurse ants. To test the nurse ants, newly frozen workers from nestmates (from the same colony) or non-nestmates (from a different colony) were used as stimuli. Following the method of Guerrieri and d’Ettorre [36], nurse workers were mildly anesthetized using CO_2_ until they stopped moving. Each nurse ant was carefully controlled to expose only its head and then placed undisturbed in a quiet location for a period of 2 h to facilitate recovery from anesthesia and acclimatization. Subsequently, their response to different stimuli was assessed. We gently touched the antennae of the nurse ant with a stimulus source for 10 s. If the duration of the nurse ant’s mandible opening exceeded 1 s, it was recorded as presence or showing a threat response, and less than 1 s was recorded as no response. Each nurse ant and stimulus worker were used only once. Seven or eight ants were tested per group. Six groups were used to replicate the assays for each treatment.

### 2.7. Data Analysis

The survival of nurse ants was analyzed using Kaplan–Meier survival analysis. The movement level (total distance, duration, and average speed of movement), brood care behavior (frequency and total time of brood care), and phototaxis (total time spend in the light zone) were analyzed using two independent sample t-test. The effects of JHA treatment and stimuli on MOR of nurse workers were analyzed using a general linear mixed effects model. Subsequently, the proportion of positive MOR responses was analyzed using two independent sample *t*-tests. All analyses were conducted using SPSS 23.0.

## 3. Results

**Figure 1:**
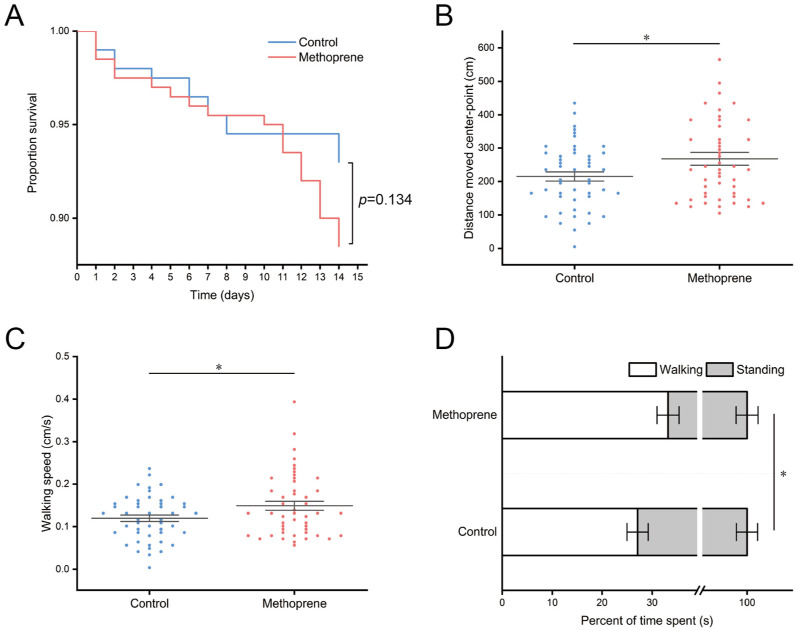
Survival and movement levels of nurse ants in methoprene treatment group and the control group. (**A**) Proportion survival. (**B**) Total movement distance. (**C**) Average movement speed. (**D**) Total movement time. “*”, *p* < 0.05.

After 14 days of feeding treatment, the survival rates of nurse ants in the JHA methoprene treatment group and control group were 88.5% and 93%, respectively (Figure 1A). There was no significant difference in survival between the two groups (*p* = 0.134). However, our results showed that treatment with JHA significantly impacts the movement level of nurse ants. Compared to the control group, the methoprene-treated group exhibited a significant increase in the total movement distance (*t* = −2.239, df = 94, *p* = 0.028) (Figure 1B), average movement speed (*t* = −2.234, df = 94, *p* = 0.028) (Figure 1C), and proportion of movement time (*t* = −1.999, df = 94, *p* = 0.048) (Figure 1D) of nurse ants.

**Figure 2:**
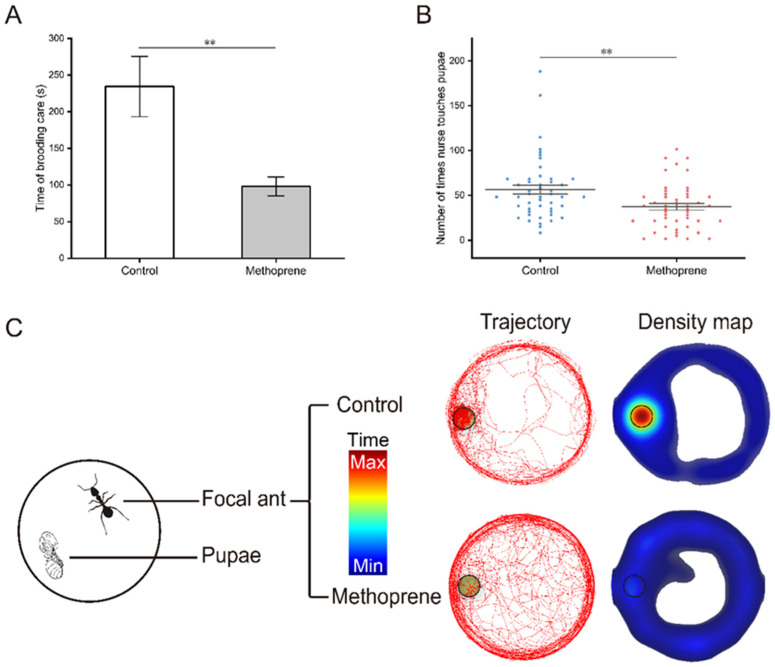
Brood care behaviors of nurse ants in the methoprene-treated group and the control group. (**A**) Total time of brooding care. (**B**) Frequency of brood care. (**C**) Movement trajectory and density map. Black circles indicate where the pupa is located. “**”, *p* < 0.01.

The video of brood care behavior was analyzed using EthoVision XT software and the movement trajectory and density map of nurse ants were obtained (Figure 2C). It can be more intuitively observed that compared to the control group, methoprene-treated nurse ants spent less time moving or staying densely around the pupa (*t* = 3.157, df = 55.924, *p* = 0.003) (Figure 2A), and there was also a significant reduction in the frequency of brood care (*t* = 3.092, df = 94, *p* = 0.003) (Figure 2B).

**Figure 3:**
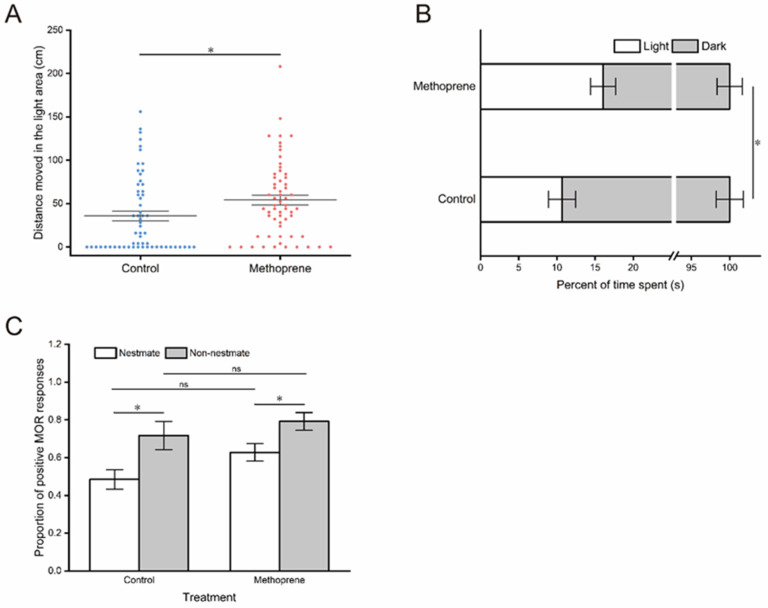
Phototactic behavior and threat responsiveness of nurse ants in the methoprene-treated group and the control group. (**A**) Movement distances in the light observation zone. (**B**) Time proportion in the light observation zone. (**C**) The proportion of positive MOR responses. “*”, *p* < 0.05; “ns”, no significant difference.

The results of the phototropic behavior test demonstrated that JHA treatment significantly enhanced the phototaxis of nurse ants, as indicated by a significant increase in the movement distance (*t* = −2.272, df = 118, *p* = 0.025) (Figure 3A) and time proportion (*t* = −2.211, df = 118, *p* = 0.029) (Figure 3B) of nurse ants in the light zone after methoprene treatment compared to the control group.

In the assay of threat responsiveness, a significant main effect of stimuli was identified (*F*_(1, 20)_ = 12.306, *p* = 0.002); however, the MOR of nurse ants was not significantly influenced by the JHA treatment (*F*_(1, 20)_ = 3.707, *p*=0.069) (Table 1). There was no significant interaction between the JHA treatment and stimuli (*F*_(1, 20)_ = 0.368, *p* = 0.551) (Table 1). Nurse ants from both the methoprene treatment group and control group exhibited a higher proportion of positive MOR responses to non-nestmate stimuli (for the control group: *t* = −2.547, df = 10, *p* = 0.029; for methoprene-treated group: *t* = −2.461, df = 10, *p* = 0.034) (Figure 3C). However, there was no significant difference in the proportion of MOR responses to the same kind of stimuli (for nestmate: *t* = −2.048, df = 10, *p* = 0.068; for non-nestmate: *t* = −0.839, df = 10, *p* = 0.421) (Figure 3C).

## 4. Discussion

Brood care is an essential behavior for social insects, which is in the interests of all colony members and represents a comprehensive reproductive investment by the colony [37]. The growth and development of ant broods are closely linked to the brood care behavior of workers [38,39]. Among several social insect species, nest workers are the main group responsible for caring for the brood and generally have a lower activity compared to foragers. However, the JH level of foraging workers is higher than that of nest workers [15,16,17,18,19], suggesting a close relationship between JH levels and brood care as well as activity behavior in social insects. The application of the JH analogue (JHA) methoprene in ant *Lasius niger* queens resulted in a significant increase in time spent active and a reduction in investment in maternal care (cleaning, guarding, or feeding) [33]. Honeybee, *Apis mellifera*, workers treated with JHA pyriproxyfen exhibited reduced brood care behavior [40]. Similarly, our results demonstrate a significant reduction in the time spent by nurse workers on pupae care and an increase in their activity level with the elevation of JH levels.

The difference in phototaxis between foraging workers and nurse workers in social insects reflects, to some extent, the differences in their division of labor. In the ant *Myrmica rubra*, the movements and attraction towards light of outside workers (foragers) are stronger than those of the inside workers (brood tenders) [41]. The transition of honeybee workers from nurses to foragers is also reflected in behavioral responses to light and circadian-rhythm-related stimuli [42]. In the bumble bee *Bombus terrestris*, foragers exhibited a stronger phototactic response compared to nurses [43]. High levels of JH titers regulate the behavior changes of social insects by inducing an increase in workers’ activities outside the nest [19]. The phototaxis of workers may also be affected during this process. The application of the JH-III analog methoprene to workers in the leaf-cutting ant *Acromyrmex octospinosus* significantly increased the amount of time spent by workers in light areas [20]. In contrast, Starkey et al. [44] demonstrated that the phototaxis of RIFA workers remains unaffected by the direct application of the JHA S-hydroprene. This observation implies a potential correlation between the impact of JHA on worker ant phototaxis and the specific type of JHA, as well as the dosage applied. Our results showed that the application of JHA methoprene increased the phototaxis of RIFA nurse workers, as evidenced by a significant increase in the activity time of nurses treated with JHA in the light area. It was revealed that the application of JHA promoted a shift in nurses’ behavior from intranidal tasks to extranidal activity.

Juvenile hormones play a role in regulating aggressive behavior in social insects [7,45], although the association is complex. In workers of honey bees, *Apis mellifera*, aggressive individuals had significantly higher JH titers than non-aggressive bees, which suggested that defense by individual bees against non-nestmates is correlated with their JH titers [7]. JH likely accelerates age polyethism in paper wasps, *Polistes dominulus* [46], and workers become more aggressive towards predators as they age [19,46,47]. The threat responsiveness of workers in social insects can reflect their aggressive behaviors and is a factor in evaluating behavioral transitions. The application of the JH analogue methoprene to *Acromyrmex octospinosus* worker ants increased their responsiveness to threats [20]. However, our findings indicate that elevated JH levels did not significantly enhance the aggressiveness of RIFA nurse workers towards stimuli (non-nestmates), possibly due to the divergent effects of JH across insect species. The aggressive behavior of *Dinoponera quadriceps* worker ants remained unaffected despite the presence of elevated JH levels [48]. In this experiment, only worker ants (nestmates or non-nestmates) were used as stimuli to evaluate the threat responsiveness of nurse workers after JHA application, while further research is needed to investigate their response to other stimuli such as brood and queen.

Research on the behavioral effects of JH on workers contributes to our understanding of the mechanisms underlying the division of labor in social insects [14,19,20]. Our study clarifies the effects of JHA on the activity levels, brood care behavior, phototaxis, and threat responsiveness of nurse workers in RIFA. Studies on the behavioral mechanisms of social insects have gradually evolved from endocrine to molecular mechanisms, and some studies have shown that gene regulation also exerts an essential influence on the behavioral transition of social insects [27,49,50]. Further investigation is still required to elucidate the molecular mechanisms underlying behavioral transitions in RIFA. In addition, the social behaviors exhibited by RIFA, including grooming, necrophoric behavior, and defense mechanisms, significantly impact the effectiveness of toxic baits as well as biocontrol agents such as fungi, viruses, nematodes, and repellent, thereby posing challenges to effective control measures [51,52,53]. This study clarifies the behavioral effects of JHA application on RIFA, demonstrating that JHA can reduce the brood care behavior of nurse workers. Our results provide an experimental basis for the application of JHA in pest control for RIFA.

## Figures and Tables

**Table 1:** Effects of JHA treatment and stimuli on MOR of nurse workers.

Source	df	Mean Square	F	*p*-Value
Treatment	1	0.071	3.707	0.069
Stimuli	1	0.235	12.306	0.002
Treatment × Stimuli	1	0.007	0.368	0.551
Error	20	0.019		
R^2^	0.450			

## Data Availability

The data presented in this study are available on request from the corresponding author.
